# How is a turbidite actually deposited?

**DOI:** 10.1126/sciadv.abl9124

**Published:** 2022-01-19

**Authors:** Zhiyuan Ge, Wojciech Nemec, Age J. Vellinga, Rob L. Gawthorpe

**Affiliations:** 1 State Key Laboratory of Petroleum Resources and Prospecting, China University of Petroleum (Beijing), Beijing 102249, China.; 2 College of Geosciences, China University of Petroleum (Beijing), Beijing 102249, China.; 3 Department of Earth Science, University of Bergen, 5007 Bergen, Norway.; 4 School of Ocean and Earth Science, University of Southampton, Southampton SO14 3ZH, UK.

## Abstract

The deposition of a classic turbidite by a surge-type turbidity current, as envisaged by conceptual models, is widely considered a discrete event of continuous sediment accumulation at a falling rate by the gradually waning density flow. Here, we demonstrate, on the basis of a high-resolution advanced numerical CFD (computational fluid dynamics) simulation and rock-record examples, that the depositional event in reality involves many brief episodes of nondeposition. The reason is inherent hydraulic fluctuations of turbidity current energy driven by interfacial Kelvin-Helmholtz waves. The experimental turbidity current, with realistic grain-size composition of a natural turbidite, used only 26 to 33% of its in-place flow time for deposition, while the remaining time went to the numerous episodes of sediment bypass and transient erosion. The general stratigraphic notion of a gross incompleteness of sedimentary record may then extend down to the deposition time scale of a single turbidite.

## Teaser

Sediment deposition from continuous turbidity current is highly discontinuous due to autogenic flow energy fluctuations.

## INTRODUCTION

Turbidity currents are subaqueous turbulent sediment-gravity flows that deliver huge volumes of sand and other clastic sediment to the deep-sea floor (*1*–*3*). These flows are discrete events, with an estimated deep-water recurrence of 50 to 650 years and volumes ranging from less than 10^5^ to more than 10^9^ m^3^. Thus, at the upper end of this range, a single turbidity current is occasionally capable of transporting more sediment than the annual global output of all rivers combined (*4*, *5*). The worldwide interest in turbidity currents and their deposits, the turbidites, is primarily for a geological understanding of deep-water turbiditic systems and their depositional facies tracts, palaeogeographic reconstruction of ancient deep-marine basins, hydrocarbon reservoir characterization, mass-flow geohazard assessment in modern deep-water environments, source-to-sink sediment budget modeling, and the modern-time delivery of plastic litter and other pollutants to the deep sea (*6*–*13*).

There is probably no other coarse-clastic depositional system on Earth that would accumulate hundreds to thousands of meters of sediment by a simple repetition of one and the same discrete rare event—a turbidity current. Yet, these systems vary enormously in their dimensions and morphodynamics (*3*, *14*), partly because the turbidity currents differ considerably in their hydraulic properties and behavior (*10*, *15*), hence the great interest in turbidites and their internal vertical succession of grain size and sedimentary structures, interpreted as the in-place record of flow behavior and evolving bedform pattern (*15*–*22*).

Two main conceptual categories of turbidity current are the surge-type (single pulse) flows, generated by an abrupt release of a fixed flow volume, and the sustained (longer-duration) flows, possibly with multiple pulses, which can stream for weeks to months and are generally attributed to a multisource release or river hyperpycnal effluent (*4*, *22*–*25*). The depositional product of surge-type flow is expected to be the classic, Bouma-type turbidite T(a)bcd accumulated with a uniformly decreasing flow power, reflected in the upward fining of grain size and similar transition from upper to lower flow-regime structure of deposited sediment (*16*, *18*, *20*, *26*, *27*).

However, there are serious reasons to doubt whether this conceptual model of a uniformly decreasing flow energy is sufficiently realistic as a guide for turbidite research. First, considerable in-place velocity fluctuations, instead of a uniform decline, have been increasingly recognized in the higher-resolution monitoring studies of steady-input uniform turbidity currents (*28*–*33*). Second, episodes of syndepositional intra-turbidite transient erosion have become invoked in the recent interpretations of turbidity current deposition based on detailed outcrop observations (*4*, *20*). Can the deposition of sediment by a uniformly fed continuous flow be in reality discontinuous?

We address this contentious issue by a high-resolution advanced computational fluid dynamics (CFD) simulation of a natural-scale, average surge-type turbidity current. Deep-sea turbidity currents are difficult to observe and monitor in nature (*15*), whereas the laboratory mini-flows of solute or extremely fine-grained dilute sediment suspension, although highly instructive, are unable to reveal the impact of some important hydraulic phenomena that depend on the flow natural-scale magnitude and sediment content (*32*, *34*, *35*). We use a natural-scale CFD simulation to obviate this insight problem and to answer the intriguing research issue.

## RESULTS

**Fig. 1.**
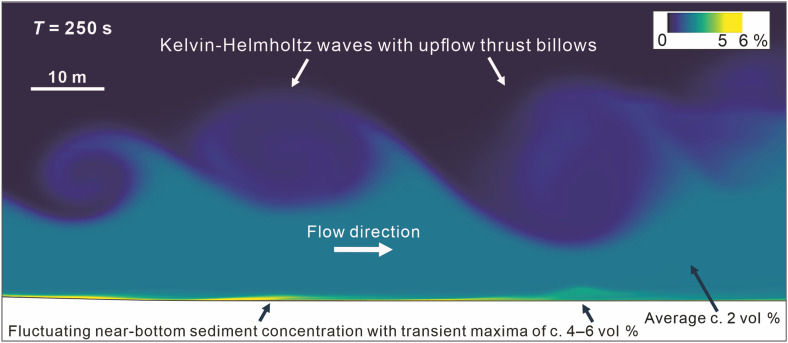
Longitudinal section snapshot of the turbidity current showing interfacial K-H waves and related bedload fluctuations. For location, see Fig. 2C.

The experimental flow at its release had immediately regulated itself by dropping excess sediment load and adjusting its hydraulics to the seafloor slope. The flow accelerated from 1.8 to 5 m s^−1^ and reduced its axial thickness from 30 to about 15 m over a travel distance of only 150 m. Vertical gradients of flow density and velocity developed, as the flow subsided by lateral expansion. Mean sediment concentration changed insignificantly, but the in-place bottom concentration considerably fluctuated (Fig. 1).

**Fig. 2.**
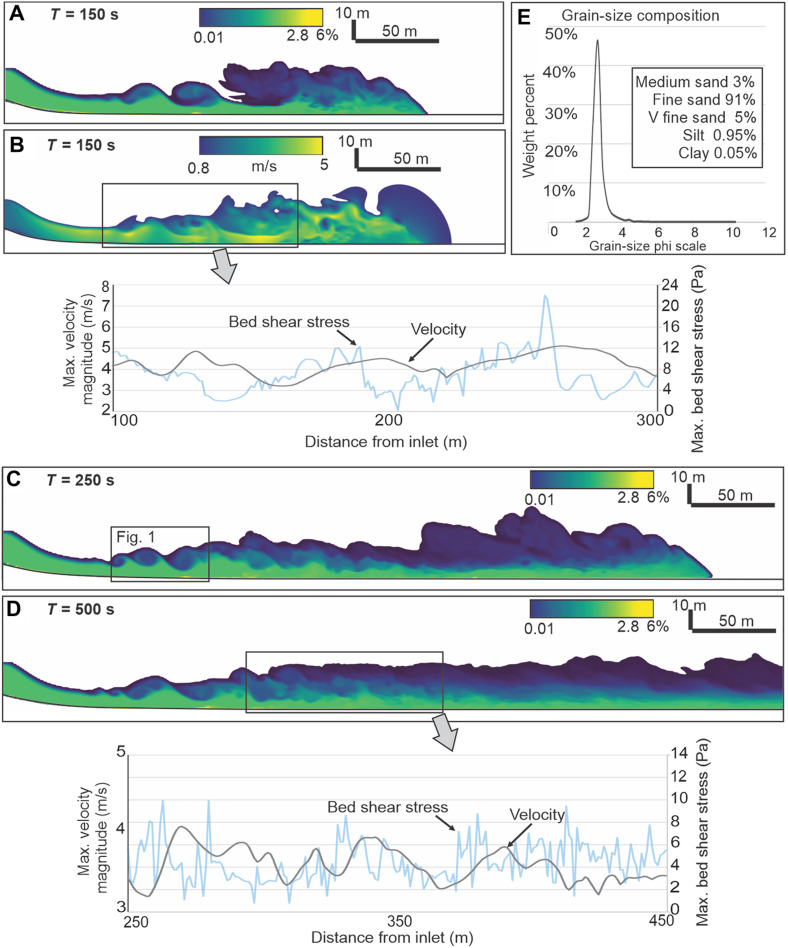
Grain-size composition and axial longitudinal display of the experimental flow. (**A**) Flow volumetric sediment concentration at 150 s after release from the gate. (**B**) Flow velocity magnitude at 150 s; the inset diagram shows in-place fluctuations of flow velocity magnitude and bed shear stress. (**C**) Flow volumetric sediment concentration at 250 s after release. (**D**) Flow volumetric sediment concentration at 500 s after release; the inset diagram shows in-place fluctuations of flow velocity magnitude and bed shear stress. Flow velocity magnitude is a geometric mean of three-dimensional velocity components and a measure of the flow in-place kinetic energy. Note in the inset diagrams that the bed shear-stress maxima follow closely the peaks of flow velocity magnitude. (**E**) Flow sediment load based on the grain-size composition of a typical turbidite from the Mount Messenger Formation, New Zealand (*53*).

As the flow in this way stabilized itself, the well-known interfacial instability in the form of Kelvin-Helmholtz (K-H) waves (*7*, *18*, *36*) commenced along its top (Fig. 1), with a wavelength of 15 to 50 m and amplitude of 5 to 10 m. This phenomenon had a major impact on the flow instantaneous velocity, with near-bed maximum magnitude fluctuating between 3 and nearly 5 m s^−1^ (Fig. 2A, inset diagram), and the bedload sediment concentration varying between 2 and 6 volume % (vol %; Fig. 1). The K-H waves were characteristically breaking and rolling upflow as billows, entraining ambient water. As the billows grew in size, they increasingly underwent random interference (Figs. 1 and 2, C and D ), and their impact on near-bed flow energy became less regular and somewhat chaotic (Fig. 2D, inset diagram).

**Fig. 3.**
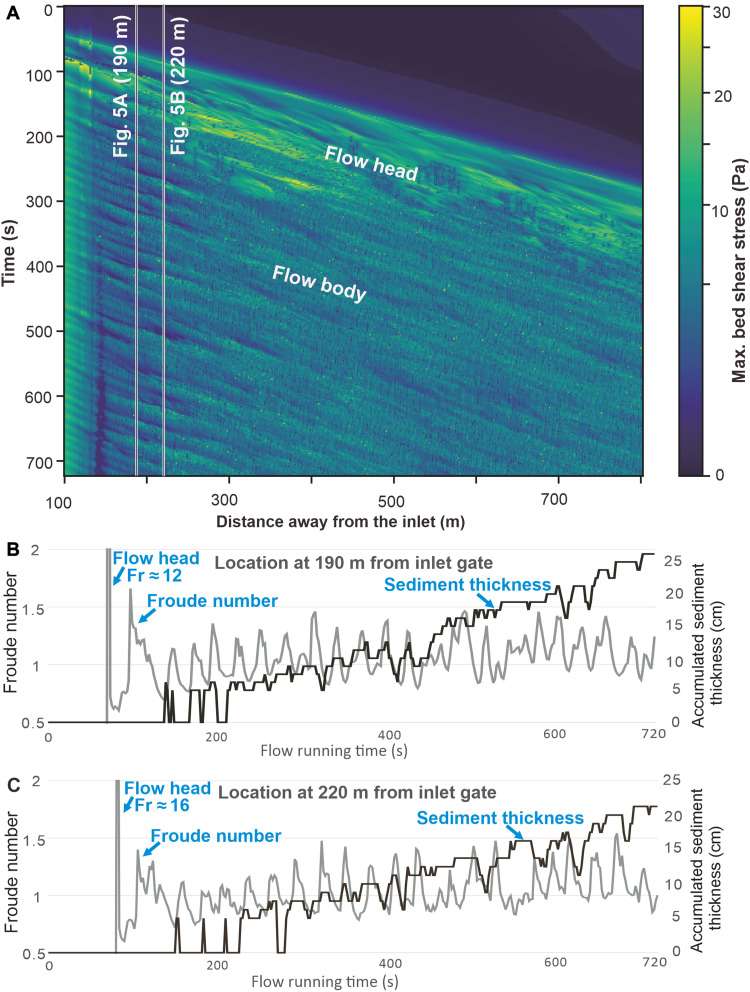
Time-series plots showing flow energy fluctuations along the axial longitudinal section of the experimental turbidity current. (**A**) Bed shear stress fluctuations of the flow head and body. (**B**) In-place fluctuations of the flow Froude number at location 190 m from inlet gate. (**C**) In-place fluctuations of the flow Froude number at location 220 m from inlet gate.

**Fig. 4.**
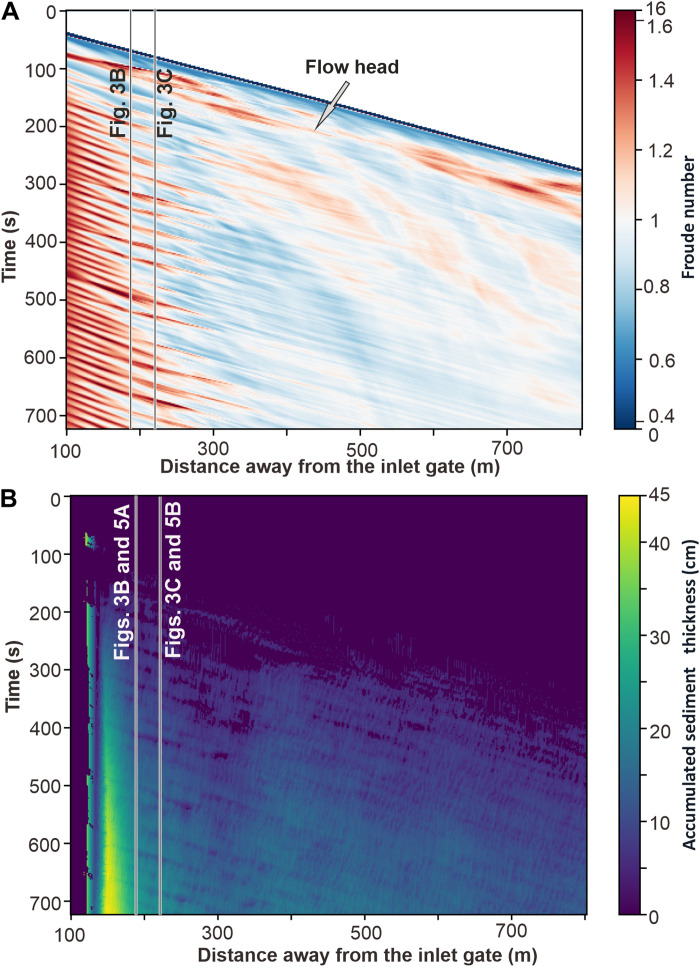
Time-series plots of Froude number and sediment thickness along the axial longitudinal section of the flow. (**A**) The Froude number changes through time over the distance of 100 to 800 m out of the inlet. Note that the legend scale is optimized for visualization. (**B**) The accumulated sediment thickness changes through time over the same distance. Note the locations of Figs. 3 (B and C) and 5 (A and B) in the plots.

An important direct hydraulic consequence of the K-H waves and energy fluctuations were the corresponding instantaneous changes of the flow bottom shear stress, which fluctuated between 0.19 and 22 Pa. The plots of sediment accumulation and bed shear stress show alternating brief episodes of sediment accretion, nondepositional bypass, and transient erosion (Fig. 2, insets). We have monitored the flow shear stress, Froude number, velocity magnitude, and sedimentary bed evolution with a time interval of 2 s (Figs. 2 to 4). The repetitive energy fluctuations (Fig. 3) are comparable to those recognized with advanced instrumental methods of natural flow monitoring (*28*, *29*, *33*), although these techniques are still incapable of showing details revealed by the present study.

**Fig. 5.**
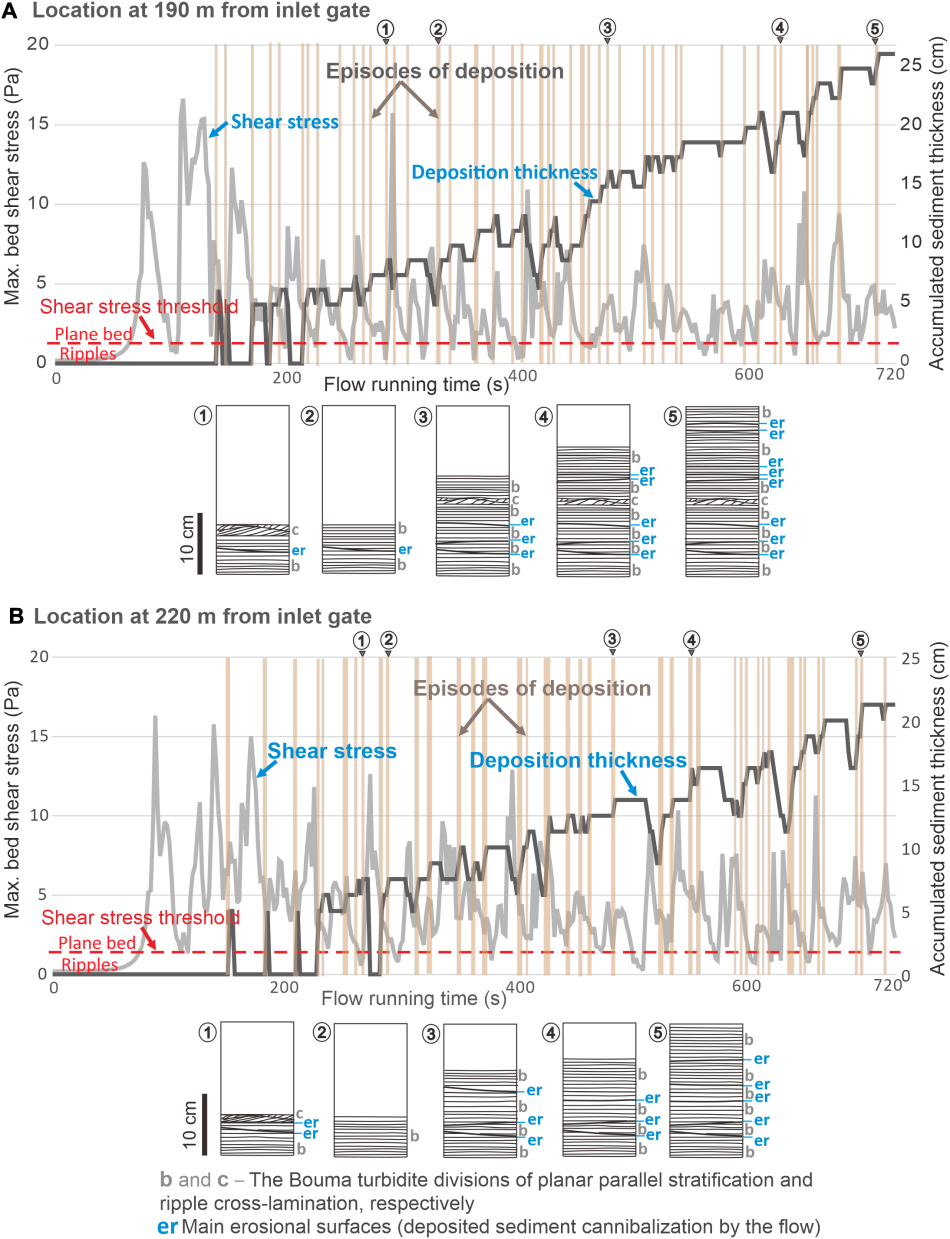
Time-series plots of bottom shear stress and sediment accumulation thickness along the axial longitudinal section of the flow. (**A**) The in-place bottom shear stress and accumulated sediment thickness at location 190 m from the flow release gate. (**B**) The in-place bottom shear stress and accumulated sediment thickness at location 220 m from the gate. The rising segments of the thickness plot (highlighted in light brown) indicate episodic deposition; the plot flat segments indicate sediment bypass and the falling segments indicate intermittent erosion. In the bottom shear-stress threshold for rippled and plane-bed transport (*37*), the stability field of unborn dunes is disregarded, and similarly ignored is the stability field for antidunes, as explained in the main text. Diagrams 1 to 5 are time snapshots of the effective vertical accretion of turbidite. Times of each diagram are shown along the top of the time-series graphs. The episodes of erosion were verified by monitoring the flow Froude number (Figs. 3, B and C , and 4A). For quantitative summary, see the main text.

For the episodes of accretion, we have predicted the mode of sediment deposition (Fig. 5, turbidite profiles) from the bedform stability diagram (*37*) based on the bottom shear stress and sediment grain size. Episodes of sediment bypass occurred when flow conditions wandered briefly into the dune bedform stability field, with the bottom shear stress too high for ripples and too low for plane-bed configuration, but with the time window too short for dune formation. Dunes are known to have a considerable time lag to form (*18*). Sediment bypass occurred also when flow conditions were hydraulically suitable for ripple formation, but the fluctuating near-bed sediment concentration happened to be insufficient. Episodes of sediment cannibalization by erosion corresponded to brief flow excursions into the supercritical regime (Froude number > 1), yet with no immediate bed defects and flowline disturbance, and with the time window too short for antidune formation (*18*, *31*).

The experimental surge-type flow formed a deposit thinning downslope from 45 to 15 cm, with notable out-of-phase changes in sediment deposition, bypass, and erosion within the monitoring distance (Figs. 3, 4B, and 5). If the flow energy was not fluctuating and a continuous deposition occurred at a uniformly decreasing rate, the deposit at the monitoring stations would be expected to be 94 to 92 cm thick (Fig. 5). The experimental flow, with its energy fluctuations driven by the K-H waves, thus effectively resulted in a deposit of about 25% of the thickness of a turbidite theoretically expected from a uniformly depositional flow surge. Around 39 to 45 episodes of in-place deposition occurred and represented only 26 to 33% of the flow time, while the remaining time went to the numerous brief nondepositional episodes of sediment bypass and erosion (Fig. 5). The deposition of sediment from a surge-type continuous flow was then highly discontinuous, and hence we conclude that the general stratigraphic notion of more gaps than depositional record (*38*) may extend down even to the deposition time scale of a single turbidite.

## DISCUSSION

### Autogenic energy fluctuations in turbidity current

The K-H waves in turbidity currents have long been considered as important for the interfacial entrainment of ambient water, but with no obvious direct impact predicted for the bedload transport (*7*, *27*). Short-frequency velocity fluctuations have been increasingly recognized in high-resolution laboratory studies of both single-pulse and multipulse turbidity currents (*25*, *28*, *30*), putting into question the notion of a “steady” flow, but their influence on the bulk dynamics of laboratory flow was considered insignificant (*25*). However, recent upscaled CFD simulations (*32*) and assessment from natural-scale flows (*33*) have indicated that the brief energy fluctuations may have a major impact on sediment transport and deposition, and that this inherent spontaneous instability of a turbidity current is virtually unrelated to a hyperpycnal river effluent and its possible pulsing (*25*, *30*). Our study confirms these important recent findings.

### Discontinuous deposition from continuous flow

The classic model of a single-pulse, surge-type turbidity current, widely invoked in textbooks, assumed a continuous deposition with no intervening episodes of sediment bypass or intrastratal erosion (*16*, *18*, *26*, *27*). Only some longer-term phenomena of net sediment bypass were considered for the nondeposition zones in certain turbiditic systems (*34*, *39*). Our study indicates that the role of intermittent bypass and erosion extends down to the internal scale of a single turbidite, with obvious important implications for the geological understanding of turbidites and the source-to-sink sediment transfer modeling studies.

**Fig. 6.**
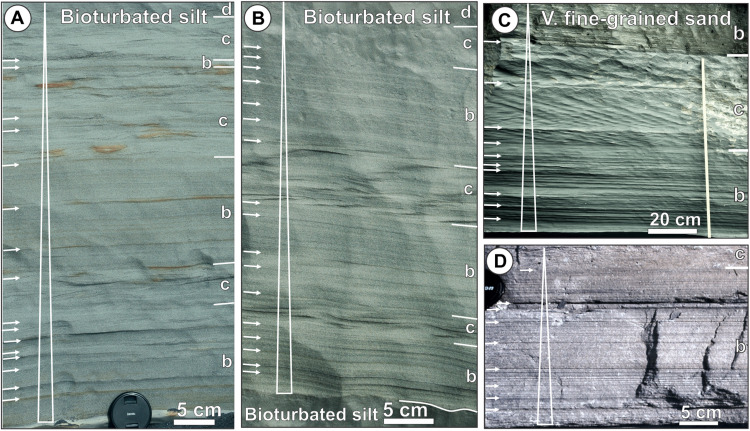
Rock-record examples of surge-type thick turbidites showing bulk normal grading (upward fining) and evidence of flow fluctuations. The examples are from (**A** to **C**) the Miocene Mount Messenger Formation, Taranaki Basin, New Zealand, and (**D**) the early Eocene Mount Jaizkibel Formation, North Pyrenean Foreland Basin, Spain. In the photographs, the white letter symbols at the right-hand margin refer to the Bouma turbidite divisions (*16*); the white arrows at the left-hand margin point to subtle and more distinct discontinuities, indicating flow in-place energy fluctuations. The elongate vertical triangles to the left indicate bulk upward fining of the sediment.

The macroscopic record of flow energy fluctuations is recognizable in turbidites, both as an alternation of their structural divisions and as intra-division discontinuities (Fig. 6). However, the idealized concept of a uniformly declining surge flow (*1*, *27*) had led to the original interpretation of every intrastratal erosional surface or upward change to higher-energy division as the base of successive turbidites. The Bouma classic sequence of turbidite divisions (*16*) was established on this premise, based on the Gres d’Annot outcrops, although many turbidites therein indicate fluctuating flows [figure 17B in (*32*)] and some even show sporadic dune cross-stratification unaccounted for in the Bouma sequence. The idealized Bouma sequence may thus not be a universal norm, but its divisions may still be useful elements for a descriptive portrayal of turbiditic deposits (*19*, *22*).

Our experimental study demonstrates that not every intrastratal erosion surface must necessarily mean a new turbidity current event, and not every fluctuation in flow energy implies a river-generated hyperpycnal flow, especially if no fluvial feeder has been evidenced. The K-H waves scale with the flow and should be distinguished from externally imposed flow pulses (*30*, *33*). Our study shows further that the flow energy fluctuations driven by K-H waves can be recorded without bedform change (Fig. 5), and may cause such a change of turbidite division only when the fluctuation occurs across the threshold boundary of bedform stability fields (*37*). Although we have used flow grain-size composition of a natural sandy turbidite, flow energy fluctuations are similarly recognizable in gravelly turbidites [figure 17C in (*32*)].

### Implications for future deep-sea research

The experimental study indicates that the depositional discontinuities in a turbidite are primarily due to the autogenic energy fluctuations in natural-scale flows, rather than to sourcing conditions or shelf regime. The issue addressed by our study is of a crucial importance to the future of turbiditic research, as the empirical recognition of substantial flow fluctuations from turbidite outcrops (*11*, *20*, *32*) has brought turbidite sedimentology to the crossroads of two conceptual trends: (i) an uncritical interpretation of all such fluctuating turbidites as river-generated hyperpycnites (*22*, *24*) or (ii) a rejection of the Bouma sequence as obsolete, with an immediate questioning of the importance of turbidity currents in theoretical favor of other submarine bottom currents (*40*, *41*). In our view, the transport and deposition by turbidity current need to be better understood before the global ubiquity and geological importance of turbiditic sedimentation can possibly be disputed.

One of the most interesting aspects of the present study is that it drastically reduces the outcrop criteria for the distinction between river-derived hyperpycnites and surge-type classic turbidites. Although the simplistic conceptual differences between the two may seem clear (*22*, *24*), the macroscopic recognition criteria are not (Fig. 6) [figure 17 in (*32*)] and require further sedimentological research.

## MATERIALS AND METHODS

The experimental turbidity current was simulated using the deterministic process-modeling commercial CFD software Flow-3D (*42*) customized for sediment gravity flows (*43*), whose reliability was verified by imitating laboratory and natural-scale turbidity currents (*44*, *45*) and which has been extensively used to simulate such underwater gravity flows (*32*, *35*, *45*). The numerical code describes fluid motion by solving the system of Reynolds-averaged Navier-Stokes equations by a finite-volume finite-difference method with the Taylor expansion (*42*) for computational grid. Flow turbulence is modeled by the renormalization group of equations with explicitly derived constants (*46*). The equations of mass and momentum conservation are time-averaged, and the turbulence model is used to account for all scales of flow vorticity (*47*). The Richardson and Zaki correlation is used in the suspension model to account for hindered settling as a function of sediment concentration. The Mastbergen and Van den Berg formulae (*48*) are used to model sediment entrainment, with the inventory of bedload transport modeled by the Meyer-Peter and Müller equation (*18*, *49*). The rate of sediment deposition is modeled by the Winterwerp *et al.* formulae (*50*). The computation grid chosen for flow axial display has a vertical resolution of 0.0125 m for bed height (sediment thickness) and 4 m for bulk flow parameters, and a horizontal resolution of 1 m. The software code allows for polysized sediment and takes account of both bathymetric pressure and Coriolis effect. In its limitations, the CFD software takes no account of sediment cohesion and of the turbulence-suppressing excess sediment concentration, considered to be dumping of bedload (*47*).

In the experiment, a nonchannelized turbidity current was released from an inlet gate 12 m wide onto a submarine slope of 20° that tangentially flattened out to horizontal over a distance of 135 m. A similar tangential seafloor topography characterizes more than 70% reported cases of a submarine slope to basin-floor transition in natural settings (*51*). The relatively steep starting slope allowed the flow to gravitationally accelerate, stabilize, and attain natural hydraulic conditions within its monitoring distance. The flow had an initial thickness of 30 m, a sediment concentration of 2 vol % (grain density, 2.65 g cm^−3^), and a velocity of 1.8 m s^−1^. The mode of flow release imitated an average surge-type turbidity current generated by localized mid-slope slumping or issued from the outlet of a modest slope channel (*15*, *36*). The initial velocity and sediment concentration were in the lower range of values calculated for natural turbidity currents (*15*), ensuring a fully turbulent flow reaching quickly a depositional mode at the transition to flat-bottom area. The flow Reynolds number was in the order of 10^5^ to 10^6^, in the upper mid-range calculated for natural turbulent flows (*10*, *15*, *27*). The inlet gate was located sufficiently far upslope to allow the flow to regulate its initial sediment load at the gate and hydraulically stabilize before reaching the monitored area of deposition. The uniform release of sediment-water mixture (total volume, 466,560 m^3^) from the inlet gate lasted 12 min. The flow monitoring along its axis was over a downslope distance of 800 m, spanning a bathymetric range of 80 to 150 m. With its tangential gradient and bathymetric range, the seafloor topography in the experiment resembled the slope of a large Gilbert-type delta or morphology of a large slump scar at shelf margin.

The sediment load carried by the current had the grain-size composition of a typical turbidite from the Miocene Mount Messenger Formation, Taranaki Basin, New Zealand (*52*), where river-derived hyperpycnal flows could be precluded and the noncemented deposits allowed for precise grain-size analyses (*53*). The unimodal and narrow-sized sediment (Fig. 2E), with a mean grain size of 0.14 mm, allowed for a high-precision predicting of sedimentary bed behavior from the diagrams of Shields threshold (*54*) and bedform stability (*37*) based on the bedload grain size and bottom shear stress. The negligible clay content avoided a high-efficiency (long runout) bypassing flow that would have to be numerically monitored for kilometers to identify its eventual switch to subcritical (depositional) mode.

To observe solely the net depositional effect of turbidity current, we assumed the seabed as nonerodible, with a surface roughness of 0.1 mm (fine sand). The assumption seemed reasonable because nonchannelized turbidity currents have little propensity for substrate erosion (*20*, *27*), and the experimental flow was dropping an excess sediment load directly outside the inlet gate, rather than attempting to erode the substrate. Furthermore, the experiment aim was to monitor the flow behavior after the onset of bedload deposition. Otherwise, the flow was fully capable of eroding and re-entraining its own deposited sediment.
