# Supplementary material for: How is a turbidite actually deposited?
Source: Sci Adv. 2022 Jan 19;8(3):eabl9124. doi: 10.1126/sciadv.abl9124 (PMC8769550; doi:10.1126/sciadv.abl9124)
Supplement: Supplementary file 1 — Supplementary Text Legends for data S1 to S8 [file sciadv.abl9124_sm.pdf]

Science Advances
AAAS

Supplementary Materials for

How is a turbidite actually deposited?

Zhiyuan Ge*, Wojciech Nemec, Age J. Vellinga, Rob L. Gawthorpe

*Corresponding author. Email: gezhiyuan@cup.edu.cn

Published 19 January 2022, *Sci. Adv.* **8**, eabl9124 (2022)
DOI: 10.1126/sciadv.abl9124

The PDF file includes:

Supplementary Text
Legends for data S1 to S8

Other Supplementary Material for this manuscript includes the following:

Data S1 to S8

Supplementary Text

This supplement contains all the data presented in the paper with the python code for the reading and displaying of some of the files.

Data S1. (separate file)

Python code for the reading and displaying of the flow Froude number, bed shear stress and accumulated sediment thickness.

Data S2. (separate file)

Data file of the flow Froude number, stored in numpy format.

Data S3. (separate file)

Data file of the maximum bed shear stress, stored in numpy format.

Data S4. (separate file)

Data file of the accumulated sediment thickness, stored in numpy format.

Data S5. (separate file)

Python code for the reading and displaying of the flow velocity magnitude and sediment concentration.

Data S6. (separate file)

Data file of the flow velocity magnitude, stored in numpy format.

Data S7. (separate file)

Data file of the flow suspended sediment concentration, stored in numpy format.

Data S8. (separate file)

Data file of the Z axis for displaying S6 and S7, stored in numpy format.
